# Reinforcement Learning for Energy Optimization with 5G Communications in Vehicular Social Networks

**DOI:** 10.3390/s20082361

**Published:** 2020-04-21

**Authors:** Hyebin Park, Yujin Lim

**Affiliations:** Department of IT engineering, Sookmyung Women’s University, Seoul 04310, Korea; hb0390@sookmyung.ac.kr

**Keywords:** 5G, D2D communication, vehicle-to-vehicle communication, mode selection, power control, vehicular social network

## Abstract

Increased data traffic resulting from the increase in the deployment of connected vehicles has become relevant in vehicular social networks (VSNs). To provide efficient communication between connected vehicles, researchers have studied device-to-device (D2D) communication. D2D communication not only reduces the energy consumption and loads of the system but also increases the system capacity by reusing cellular resources. However, D2D communication is highly affected by interference and therefore requires interference-management techniques, such as mode selection and power control. To make an optimal mode selection and power control, it is necessary to apply reinforcement learning that considers a variety of factors. In this paper, we propose a reinforcement-learning technique for energy optimization with fifth-generation communication in VSNs. To achieve energy optimization, we use centralized Q-learning in the system and distributed Q-learning in the vehicles. The proposed algorithm learns to maximize the energy efficiency of the system by adjusting the minimum signal-to-interference plus noise ratio to guarantee the outage probability. Simulations were performed to compare the performance of the proposed algorithm with that of the existing mode-selection and power-control algorithms. The proposed algorithm performed the best in terms of system energy efficiency and achievable data rate.

## 1. Introduction

With the dynamic increase in data traffic in connected vehicles and wireless networks, satisfying cellular data traffic has become relevant. To satisfy these requirements, vehicular social networks (VSNs) have been studied [1,2]. VSNs consist of moving and parked vehicles on roads, and the vehicles communicate with nearby vehicles or infrastructure to report and exchange traffic information. Therefore, one of the challenges is to ensure communication quality and reduce delays in VSNs. To guarantee a high data rate in a VSN, base stations are densely deployed and overlapped. However, densely deployed base stations cause higher system energy consumption. To tackle the energy consumption problem, the traditional base station structure is changed to a centralized structure, such as a heterogeneous cloud radio access network (H-CRAN). In the H-CRAN structure, a traditional base station is divided into a signal-processing part as the baseband unit (BBU) and a signal transceiver part as the radio remote head (RRH). The BBUs are centralized as a BBU pool, and the RRHs are connected to the BBU pool through fronthaul links. The macro base station only serves voice services to users, and the RRHs are densely overlapped in macro cells.

In traditional communication in vehicular networks, vehicles communicate with other vehicles and infrastructure through RRHs. Further, vehicles have high mobility; therefore, they make frequent handovers. This causes heavy loads and energy consumption problems in the system. To solve the energy consumption problem and provide efficient communication between vehicles, device-to-device (D2D) communication has been developed. With D2D communication, nearby wireless devices can communicate directly without an RRH, which can reduce the system energy consumption from the RRH transceivers and fronthaul links. In vehicular networks, D2D communication can facilitate efficient data communication between nearby vehicles. Thus, D2D communication can decrease latency because of the relatively small distance between the transmitter and receiver vehicle. As D2D communication reuses cellular wireless resources, it helps increase the spectral capacity.

However, D2D communication is highly susceptible to interference because of the reuse of cellular resources. Specifically, for D2D communication in H-CRANs, various interference problems are caused by the dense deployment of RRHs. In addition, when traffic congestion occurs in vehicular environments, the high density of devices in D2D communication can cause a critical interference problem [3]. To avoid the interference problem, channel extensions are conducted. However, interference problems remain because of interference from adjacent channels and shared resources [4,5,6]. To solve these problems, various studies on D2D communication have been conducted. Mode selection is a technique that can solve the interference problem [7,8,9]. Mode selection allows the devices to select from several communication modes: cellular mode, D2D mode, and detection mode. With mode selection, devices choose modes according to the channel state, which can ensure sufficient quality of service (QoS) for the devices and increase the system capacity. Another solution to solve the interference problem is to use the power-control method [10,11]. Power control is a method that manages interference by controlling the power of the D2D links. Using the power-control method increases the energy efficiency of a device by decreasing the transmission power in low-interference situations and increasing the transmission power in high-interference situations to guarantee the QoS.

In this paper, we propose a joint mode-selection and power-control algorithm using reinforcement learning in a VSN. In our algorithm, the BBU pool uses centralized Q-learning, and the vehicles use distributed Q-learning to achieve improved signal-to-interference noise ratios (SINRs). Centralized Q-learning aims to maximize the system’s energy efficiency, while distributed Q-learning aims to maximize the vehicle’s achievable data rate. As the energy efficiency of the system and the SINR of a vehicle are in a trade-off relationship, it is important to identify a point that optimizes both objectives. Our algorithm ensures vehicular capacity by considering outage probability as a QoS constraint while maximizing system energy efficiency.

The rest of this paper is organized as follows. In Section 2, we introduce related work. In Section 3, we formulate the system model and the problem definition. The joint mode selection and power-control algorithm using multi-Q-learning is introduced in Section 4. A performance evaluation and discussion of our algorithm are presented in Section 5. We present the conclusions of this study in Section 6.

## 2. Materials and Methods

To reduce the load and increase the system’s energy efficiency, several optimization methods have been developed. In [12], a mode-selection algorithm was developed to maximize the energy efficiency of the device using the transmission rate as a QoS requirement. The mode was determined adaptively, based on various factors of the device, and it could minimize the energy consumption for each successful content delivery. In [13], a mode-selection algorithm that considered the link quality of D2D links was proposed to maximize the system throughput. It estimated the expected system throughput considering the SINR and available resources, so it could maximize the system throughput.

As D2D communication reuses cellular resources, interference management between cellular and D2D links is important. The power-control method that controls the transmission power of D2D links is one method used to manage interference. In [14], a power-control algorithm with variable target SINRs was proposed for application in multicell scenarios. It aimed to maximize the system spectral efficiency using a soft-dropping algorithm to control the transmission power to meet the variable target SINR. A power-control algorithm using stochastic geometry was proposed in [15]. The algorithm can be divided into two types: centralized and decentralized. The centralized type aims to guarantee the coverage probability by solving the optimization power problem. The decentralized type was an interference mitigation method to maximize the sum rates of the devices.

To take advantage of both mode selection and power control, jointly designed algorithms were proposed. In [16], an energy-aware joint power-control and mode-selection algorithm was proposed to minimize the power consumption. It solved the power minimization problem by guaranteeing the QoS constraints and developed a joint strategy under the condition of imperfect channel state information. In [17], a mode-selection and power-control algorithm was proposed to maximize the sum of the achievable data rate. It selected the mode to satisfy the distance and interference constraints from an operator perspective. After mode selection, it first proved that the power-control problem was quasiconvex for the D2D mode and then solved it.

Environments where cellular and D2D modes coexist involve high complexity that reflects the numerous features of network dynamics in the optimization methods. To consider network dynamics, reinforcement learning was used to adapt the optimization of the mode selection and power-control problems. In [18], a mode-selection algorithm based on Q-learning was proposed to maximize QoS and minimize interference. It considered the delay, energy efficiency, and interference to determine the transmission mode. In [19], a mode-selection method based on deep reinforcement learning was proposed to minimize the system power consumption in a fog radio access network. To make optimal decisions, it formulated the energy minimization problem with a Markov decision process by considering the on-off state of processors, communication mode of the device, and precoding vectors of RRH.

Reinforcement learning was implemented in [20] to adapt the power-control algorithm for D2D communication. It consisted of centralized and distributed Q-learning and aimed to maximize the system capacity and guarantee a stable QoS level. In centralized Q-learning, called team Q-learning, the agent in each resource block (RB) managed only one Q-table. In distributed Q-learning, agents in each D2D link learned independently and managed each Q-table. Team Q-learning could reduce the complexity and avoid the overhead from managing the Q-tables in distributed Q-learning. In [21], a joint mode-selection and power-control algorithm with multiagent learning was proposed. The agents in each device managed each Q-table and learned independently. It considered the local SINR information and device modes, such as the cellular mode, D2D mode, and detection mode. It helped D2D transmitters decide on efficient mode selection and power control to maximize the energy efficiency of the D2D links.

In a VSN, vehicles move constantly with high mobility. This high mobility results in frequent handovers in cellular communication and changes the channel states accordingly. However, none of the above studies considered vehicle mobility and changing channel states. In addition, there is a large amount of communication between adjacent vehicles in a VSN. D2D communication has distance limitations that have a significant impact on performance; thus, vehicle mobility should be considered. Therefore, any appropriate mode-selection and power-control method must consider the network dynamics that come with vehicle mobility. This mobility causes signaling overhead and data latency because of frequent changes in the channel information state.

Typical optimization methods incur high complexity if they consider the various relevant features. To make optimal decisions in a dynamic network environment, reinforcement learning can make recommendations according to the various states. In D2D communication applications, deep Q-learning has the advantage of being able to learn directly using network data and process high-dimensional data [15]. However, deep Q-learning is more complex than Q-learning, and the available data for vehicles are limited. So, it brings high complexity and overhead problems.

In this paper, we propose a mode-selection and power-control algorithm using reinforcement learning for the H-CRAN architecture in a VSN. Our algorithm consists of two parts: centralized Q-learning and distributed Q-learning. In the centralized part, the agent in a BBU pool manages one Q-table to maximize the system’s energy efficiency and guarantee the QoS constraints. It recommends an appropriate communication mode and transmission power for the vehicles, based on the average SINR and available resources. To satisfy the outage probability as a QoS constraint, the target SINR that determines the states is adjusted. In the distributed part, the agents in each vehicle manage each table and learn to maximize the received SINR. The agents choose their actions by comparing the actions recommended by the BBU with their own actions.

## 3. System Model and Problem Definition

**Figure 1:**
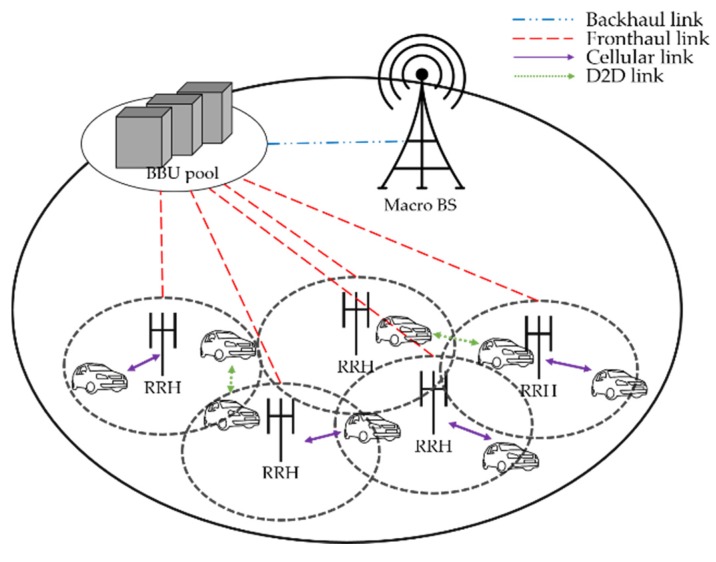
System model.

In this work, we consider the single-cell scenario in H-CRANs where cellular-mode vehicles and D2D-mode vehicles coexist. The vehicles are expressed as devices in this study. H-CRANs comprise a BBU pool and multiple RRHs, as shown in Figure 1. The total device set can be expressed as U, and it consists of a cellular device set C and D2D device set D. The cellular devices, C={1,…,c}, and the number of D2D devices are distributed randomly within the set D={1,…,d}. We denote the set of RRHs as S={1,…,s} and RBs as K={1,…,k}. We assume that one RB can be allocated to one cellular device and shared with multiple D2D devices. Each device can select the transmission mode between the cellular mode, $m_{c}$, and D2D mode, $m_{d}$; to select the D2D mode, the distance between the D2D pairs must satisfy the D2D distance threshold.

The SINR of cellular device c at the k-th RB, as reported in [20], is

(1) $$\gamma_{k}^{c}=\frac{p_{tr}^{0}\cdot g_{k}^{sc}}{N+\sum_{d\in D}p_{k}^{d}\cdot g_{k}^{cd}+\sum_{s^{\prime }\in RRH,\;s^{\prime }\neq s}p_{tr}^{0}\cdot g_{k}^{s^{\prime }c}\;},$$

where $p_{tr}^{0}$ is the transmission power of the RRH; $g_{k}^{sc}$ is the channel gain between the associated RRH s and cellular device c, and N is the noise power spectral density. The channel gain between cellular device c and the associated RRH s can be calculated as $g_{k}^{sc}=G\varrho^{s,c}\xi^{s,c}L^{s,c}{}^{-ο}$, where G is the constant gain from the antenna and amplifier, $\varrho^{s,c}$ is the multipath fading gain with a log-normal distribution, $L^{s,c}$ is the distance between the cellular device c and the associated RRH s, and ο is the path-loss exponent. The SINR of D2D device d at the k-th RB, as reported in [16], is

(2) $$\gamma_{k}^{d}=\frac{p_{k}^{d}\cdot g_{k}^{dd^{\prime }}}{N+p_{k}^{c}\cdot g_{k}^{cd}+\sum_{j\in D,\;j\neq d}p_{k}^{j}\cdot g_{k}^{jd}\;},$$

where $p_{k}^{d}$ is the transmission power of D2D device d, $g_{k}^{dd^{\prime }}$ is the channel gain between another D2D pair $d^{\prime }$, $p_{k}^{c}$ is the transmission power of cellular device c which shares the k-th RB, and $g_{k}^{cd}$ is the channel gain between cellular device c and D2D device d. The total system capacity, according to the Shannon capacity, can be expressed as

(3) $$Β=W^{total}\sum_{c\in C}\sum_{k\in K}\left\{\log_{2}\left(1+\gamma_{k}^{c}\right)+\sum_{d\in D}\log_{2}\left(1+\gamma_{k}^{d}\right)\right\},$$

where $W^{total}$ is the total bandwidth of the system. The power consumption of the system includes the power consumed by the RRHs and fronthaul devices. The power consumption model of the RRH can be expressed as

(4) $$P_{rrh}=\sum_{s=1}^{S}\left(\phi_{rrh}+\Delta slope\sum_{c=1}^{C}a_{c}^{s}p_{tr}^{0}\right),$$

where $\phi_{rrh}$ is the circuit power of the RRH, Δslope is the slope of the load-dependent power consumption of RRH, as reported in [22], and $a_{c}^{s}$ is the association indicator of cellular device c, with values of 1 for an association and 0 for a nonassociation. The power consumption model for the fronthaul links was reported in [23] and can be expressed as

(5) $$P_{fronthaul}=\sum_{s=1}^{S}\left(\phi_{fronthaul}+\ell \cdot t_{s}\right),$$

where $\phi_{fronthaul}$ is the circuit power from the fronthaul transceiver and switch, ℓ is the power consumption per bit/s, and $t_{s}$ is the traffic associated with RRH s. The macro base station provides only voice services; thus, its power consumption is not considered. Therefore, the system power consumption model can be defined as

(6) $$P=P_{rrh}+P_{fronthaul}.$$

The system energy efficiency can be defined as

(7) $$EE=\frac{B}{P}.$$

Our main goal is to maximize the system’s energy efficiency, and it can be formulated as

(8) $$\underset{C,D,K}{\max}EE,$$

$$\begin{array}{c} s.t.C1:\gamma \geq \gamma_{0}\forall c,d,k,\\ C2:0<p_{k}^{d}\leq p_{max}\forall d,k,\\ C3:\tau \leq \tau_{max}. \end{array}$$

where γ is the SINR of users, $\gamma_{0}$ is the SINR constraint, $p_{max}$ is the maximum transmission power, τ is the outage probability, and $\tau_{max}$ is the maximum outage probability constraint. The outage probability is the probability that the SINR of the devices is lower than the SINR constraint [24].

## 4. Proposed Algorithm

In this section, we introduce a mode-selection and power-control algorithm based on Q-learning. When each device has Q-learning agent, the agent cannot get the information to improve system energy efficiency at system level. Even if the system sends the information to the agent, it increases that state space that agent manages and the communication load for data exchange between system and device. So, we proposed two types of Q-learning agent: centralized agent at the system level and distributed agent at each device.

In our previous research, we proposed RRH switching and power-control methods based on the Q-learning mechanism [25]. We considered the available resources and interference levels to maximize the energy efficiency and minimize interference in the cell. However, this cannot account for the QoS of the devices, and cell coverage problems occur because of switching off the RRHs. We need to ensure the QoS of the devices while maximizing the system’s energy efficiency, which is in a trade-off relationship. Therefore, in this paper, we determine communication mode and transmission power by using Q-learning mechanism to solve the problems. The centralized agent learns to recommend optimal actions that can maximize system energy efficiency by considering the available resources and interference in the cell. The distributed agent recommends an optimal action that can maximize the SINR of the device by considering the interference of the device. Then, the agent finally selects the optimal action by comparing the expected SINRs of the recommended actions.

Existing algorithms have focused on maximizing the energy efficiency of the devices, which cannot guarantee QoS. Our proposed algorithm learns to maximize the system’s energy efficiency and the received SINRs of devices. It also adjusts the target SINR, which is the basis for the proposed Q-learning, according to the interference state. It is important to set the target SINR appropriately because as the target SINR increases, the agent increases the transmission power to increase the SINR. This increases the cellular mode and reduces the system’s energy efficiency. Conversely, when the target SINR is reduced, the agent reduces the transmission power to lower the SINR. This increases the system’s energy efficiency, but it is likely that the devices cannot guarantee the QoS. The agents of each device are in the transceiver of each device, and we assume that agents get the information of the receiver through delay-free feedback [26].

The operational procedure is described as follows. In Step 1, the device prepares to select the transmission mode. At this step, the transmitting device requests a connection through the BBU pool to communicate with the receiving device, and the BBU pool checks whether the distance between the devices is under the D2D distance threshold. If the device does not satisfy the D2D distance threshold, the device will only be able to select the cellular mode and associate with the RRH that provides the highest SINR. If the device satisfies the D2D distance threshold, it can select the D2D mode and obtain the selectable mode and transmission power from its agent. After that, the BBU pool recommends the selectable mode and transmission power to the device.

**Figure 2:**
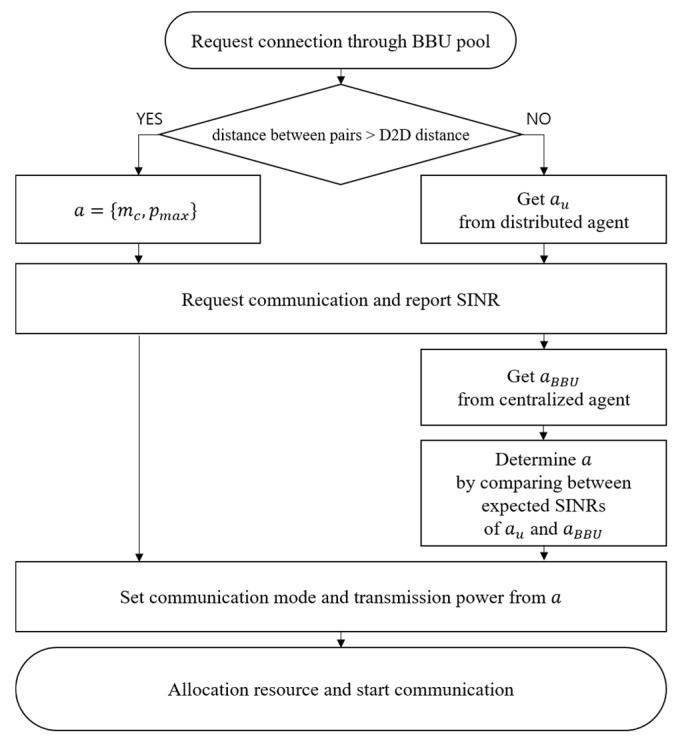
Operation procedure.

In Step 2, the agent calculates the expected SINRs of the recommended actions from its own agent and from the BBU pool. The agent chooses communication mode and transmission power as its action. The agent then informs the BBU pool of the determined action. The BBU pool allocates resources to the device, and the device starts communication based on the determined actions. This procedure is summarized in Figure 2.

To recommend the communication mode and transmission power, Q-learning is used in the BBU pool and in each device. Q-learning is a model-free reinforcement-learning algorithm that learns to find an optimal policy that can maximize the expected reward. Q-learning involves a set of states S, a set of actions A, and a set of rewards R. The agent transits from one state to another state by performing an action a. The agent chooses an action according to the optimal policy $\pi^{*}$ in its current state s. The agents manage the Q-table, and the Q-value updating rule is defined as follows:

(9) $$Q^{t+1}\left(s^{t},a^{t}\right)=Q^{t}\left(s^{t},a^{t}\right)+\alpha \left[r^{t+1}+\beta \underset{a}{max}Q^{t}\left(s^{t+1},a^{t+1}\right)-Q^{t}\left(s^{t},a^{t}\right)\right],$$

where $Q^{t+1}\left(s^{t},a^{t}\right)$ is the Q-value with state $s^{t}$ and action $a^{t}$ at time t, α is the learning rate, $r^{t+1}$ is the expected reward at time t+1, and β is the discount factor. The optimal policy $\pi^{*}$ with state s can be expressed as

(10) $$\pi^{*}\left(s\right)=\underset{a}{max}Q\left(s,a\right).$$

### 4.1. Centralized Q-Learning in the BBU Pool

To recommend the communication mode and transmission power, based on Equation (8), we use a Q-learning agent in the BBU pool. For Q-learning in the BBU pool, the state, action, and reward can be defined as follows. The state of the BBU pool at time t is defined as

(11) $$S_{BBU}^{t}=\left\{I^{t},\;\rho^{t}\right\},$$

where $I^{t}$ is the binary level of the average received SINR of the devices at time t, with a value of 1 when the average SINR is greater than the target SINR, $\gamma_{min}$, and 0 when the average SINR is smaller than $\gamma_{min}$. $\rho^{t}$ is the available RB at time t. The action of the BBU pool at time t is defined as

(12) $$A_{BBU}^{t}=\left\{m^{t},p^{t}\right\},$$

where $m^{t}$ is the transmission mode at time t, and $p^{t}$ is the transmission power at time t. The transmission power $p^{t}$ is divided into discrete intervals, $\left(0,\;p_{max}\right]$. If the transmission mode $m^{t}$ is the cellular mode $m_{c}$, the transmission power $p^{t}$ will be set to $p_{max}$. The reward of the BBU pool at time t is defined as

(13) $$R_{BBU}^{t}=EE.$$

The centralized Q-learning is summarized in Algorithm 1.
**Algorithm 1: Pseudocode for centralized Q-learning**Initialization: for each $s_{BBU}^{t}\in S_{BBU}^{t},\;a_{BBU}^{t}\in A_{BBU}^{t}$ do  initialize Q-table and policy $\pi_{BBU}^{*}\left(s_{BBU}^{t}\right)$ end forLearning: loop  estimate the state $s_{BBU}^{t}$  generate a random real number *x*∈[0,1]   if x<ε // for exploration    select the action $a_{BBU}^{t}$ randomly   else    select the action $a_{BBU}^{t}$ according to $\pi_{BBU}^{*}\left(s_{BBU}^{t}\right)$   recommend action $a_{BBU}^{t}$ to the devices in the cell   calculate reward $r_{BBU}^{t}$   update Q-value $Q\left(s_{BBU}^{t},\;a_{BBU}^{t}\right)$ and $\pi_{BBU}^{*}\left(s_{BBU}^{t}\right)$end loop

### 4.2. Distributed Q-Learning in the Devices

According to Equation (8), the BBU pool recommends more D2D modes for the devices. While the D2D mode has the advantage of increasing system capacity, it may not ensure an achievable data rate because of interference. To solve this problem, we use Q-learning agents in each device to maximize the received SINR.

The state of device u at time t is defined as

(14) $$S_{u}^{t}=\left\{I_{u}^{t}\right\},$$

where $I_{u}^{t}$ is the binary level of the received SINR of device u at time t based on current communication mode. If the mode of device is cellular mode, state of device u will be calculated based on received SINR of the cellular link. The action of device u at time t is defined as

(15) $$A_{u}^{t}=\left\{m_{u}^{t},\;p_{u}^{t}\right\},$$

where $m_{u}^{t}$ is the transmission mode of device u at time t, and $p_{u}^{t}$ is the transmission power of device u at time t. The reward of device u at time t is defined as

(16) $$R_{u}^{t}=\{\begin{array}{c} \gamma_{u}\;\;\;\;\;\;\;,I_{u}^{t}=1\\ -\gamma_{u}*\left(p_{max}-p_{u}^{t}\right),I_{u}^{t}=0,\;\gamma_{u}\geq 0\\ \gamma_{u}*\left(p_{max}-p_{u}^{t}\right)\;,I_{u}^{t}=0,\;\gamma_{u}<0 \end{array},$$

where $\gamma_{u}$ is the SINR of device u. In distributed Q-learning, the agent chooses between the action from centralized Q-learning and its own action to find the action that provides a higher expected SINR. Distributed Q-learning is summarized in Algorithm 2.
**Algorithm 2: Pseudocode for distributed Q-learning**Initialization:  for each $s_{u}^{t}\in S_{u}^{t},\;a_{u}^{t}\in A_{u}^{t}$ do   initialize Q-table and policy $\pi_{u}^{*}\left(s_{u}^{t}\right)$
 end for Learning:  loop   estimate state $s_{u}^{t}$
  generate a random real number *x*∈[0,1]
  if x<ε // for exploration    elect action $a_{u}^{t}$ randomly   else    select action $a_{u}^{t}$ according to $\pi_{u}^{*}\left(s_{u}^{t}\right)$
  receive action $a_{BBU}^{t}$ from algorithm1   determine action $a_{u}^{*}$ by comparing $a_{u}^{t}$ and $a_{BBU}^{t}$  execute action $a_{u}^{t}$
  calculate reward $r_{u}^{t}$
  update Q-value $Q\left(s_{u}^{t},\;a_{u}^{t}\right)$ and $\pi_{u}^{*}\left(s_{u}^{t}\right)$
end loop

### 4.3. Target SINR Updating Algorithm

In our algorithm, the state of Q-learning is determined by the target SINR. To set the target SINR to reflect the interference state, the target SINR at time interval T, denoted as $\gamma_{min}^{T}$, is adjusted as follows:

(17) $$\gamma_{min}^{T+1}=\{\begin{array}{cc} ∁*\gamma^{+}+\left(1-∁\right)*\gamma_{min}^{T}, & \tau^{T}\geq \tau_{max}\;\\ ∁*\left(\frac{\gamma_{median}+\gamma_{0}}{2}\right)+\left(1-∁\right)*\gamma_{min,}^{T} & \tau^{T}\leq \tau_{min} \end{array},$$

where ∁ is the weight factor, $\gamma^{+}$ is the largest SINR value of the devices, $\tau^{T}$ is the average outage probability for the time interval T, and $\tau_{max}$ is the maximum outage probability. $\gamma_{median}$ is the median SINR value of the devices; $\gamma_{0}$ is the SINR constraint, and $\tau_{min}$ is the minimum outage probability.

The changed target SINR affects the learning, and therefore, the agent can make an optimal decision using the changed target SINR. However, as the optimal actions in the changed target SINR may not be optimal in the original target SINR, those actions may not be selected. To solve this problem, we must adjust the Q-table. At this time, only the Q-table of the BBU is adjusted as follows:

(18) $$Q^{T+1}\left(s,a\right)={log}_{2}\left(Q^{T}\left(s,a\right)-1\right)\;\forall \left\{s,a\right\}.$$

## 5. Results and Discussion

A single-cell H-CRAN environment was considered in this work, and the parameters used in the simulation are summarized in Table 1. We set the parameters and speed requirements for mobility dataset according to the 3GPP (3rd Generation Partnership Project) specifications release 16 [22,27]. Four RRHs and vehicles were distributed randomly in a single macro cell with an intercell distance of 500 m. The mobility dataset used in the simulation was a dataset in an urban area created using a simulation of urban mobility (SUMO) simulator [28]. In the dataset, all vehicles had mobility with a random trip model at the Seoul City Hall in South Korea according to the 3GPP specification release 15 [29]. The datasets consisted of two types of scenarios: light traffic and heavy traffic. The two types were vehicular mobility datasets for urban areas with light traffic and heavy traffic scenarios. In a light traffic scenario, vehicles can move fast and the distance between vehicles increases. This means that fewer vehicles can select the D2D mode because of the D2D distance threshold. In a heavy traffic scenario, vehicles move slowly because of the traffic jam, and the distance between vehicles becomes shorter. This allows the vehicles to select a D2D mode more often. Compared to heavy traffic scenarios, fewer vehicles move and require resources at the same system load situation in light traffic scenarios. The D2D distance threshold affects the system energy efficiency, which can also be affected by the mode selection. To consider and present the effects of the D2D distance threshold, we simulated experiments with various D2D distances, as reported in [27]. We considered Rayleigh fading, log-normal shadowing, and the path-loss model 140.7+36.7log(distance(km)), based on [30]. The D2D distance threshold was varied between 250 and 350 m, according to [31,32]. The Q-learning parameters were α=0.01, β=0.9, and ε=0.01. We set the time for the D2D link establishment as 6 ms, as reported in [33], and the size of time unit is 3 s.

We performed simulations while changing the load of the system and the D2D distance with different traffic scenarios and compared the proposed algorithm with other existing algorithms. First, we denote our proposed algorithm as “proposed algorithm”. Second, the condition where no algorithm was applied was used to create a baseline, and we denoted the baseline as “BA”. It selects the communication mode by comparing the strength of the expected SINR between cellular and D2D modes. Third, for a comparison with a mode-selection and power-control algorithm with Q-learning, the algorithm in [21] was used. It learned to maximize the device’s energy efficiency with a fixed target SINR. The objective that maximizes device energy efficiency can also maximize system energy efficiency by selecting more D2D modes. We denote that as “Compare1”. Fourth, we used the algorithm in [20] to compare power control with Q-learning. It also learned to maximize device energy efficiency using a fixed target SINR. We denote that as “Compare2”.

**Figure 3:**
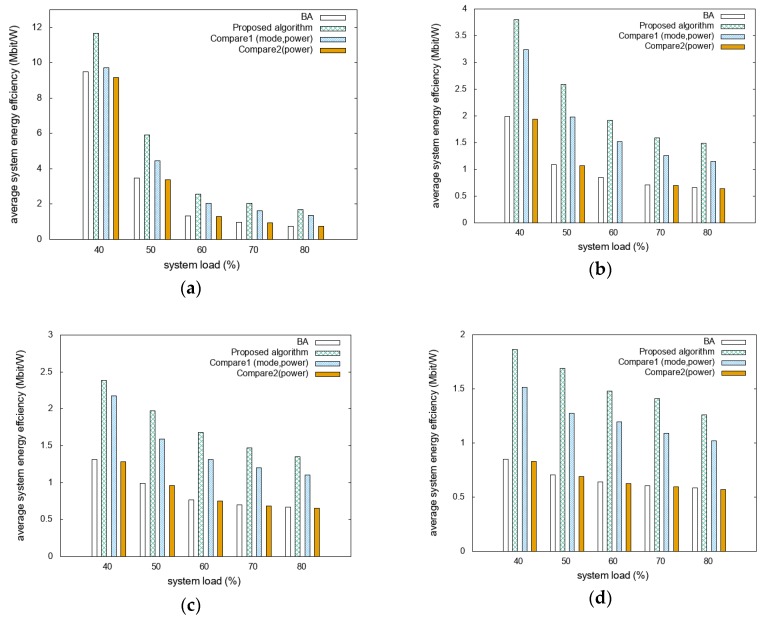
Comparison of system energy efficiencies with various device-to-device (D2D) distances and system loads: (**a**) 250 m with light traffic; (**b**) 350 m with light traffic; (**c**) 250 m with heavy traffic; and (**d**) 350 m with heavy traffic.

The energy efficiencies for different D2D distance scenarios are compared as functions of the system loads with different mobility scenarios, as shown in Figure 3. Figure 3a shows that the system energy efficiency of the proposed algorithm is 83% and 89% higher than those of BA and Compare2, respectively. Compared to Compare1, it presents high energy efficiency, with a difference of approximately 26%. In Figure 3b it can be seen that the proposed algorithm outperformed BA, Compare1, and Compare2 by more than 121%, 26%, and 126%, respectively. Figure 3c shows that the system energy efficiency of the proposed algorithm is 102% and 108% higher than that of BA and Compare2, respectively. Compared to Compare1, it presents higher energy efficiency, with a difference of approximately 21%. Figure 3d shows that the proposed algorithm performed approximately 128%, 27%, and 133% better than BA, Compare1, and Compare2, respectively. As the number of devices capable of D2D communication increases as the D2D distance increases, the difference in system energy efficiency increases. This is because the devices communicate directly rather than through the RRH, which reduces the system’s energy consumption. The proposed algorithm is designed to maximize system energy efficiency; thus, it gives the best performance in scenarios with large D2D distances. Furthermore, because D2D-mode vehicles communicate with the same transmission power at different distance thresholds, the achievable data rate and system energy efficiency decrease at longer D2D distance thresholds. This reveals that the overall performance decreases as the D2D distance threshold increases, but the proposed algorithm achieves a better performance compared to other algorithms. In addition, system energy efficiencies in the light traffic scenario get higher-scale results than the heavy traffic scenario. This is because vehicles require more resources in the light traffic scenario compared to the same number of vehicles in a heavy traffic scenario. Required resources affect system capacity, so system energy efficiencies in the light traffic scenario are higher than the heavy traffic scenario in the same system load situation.

**Figure 4:**
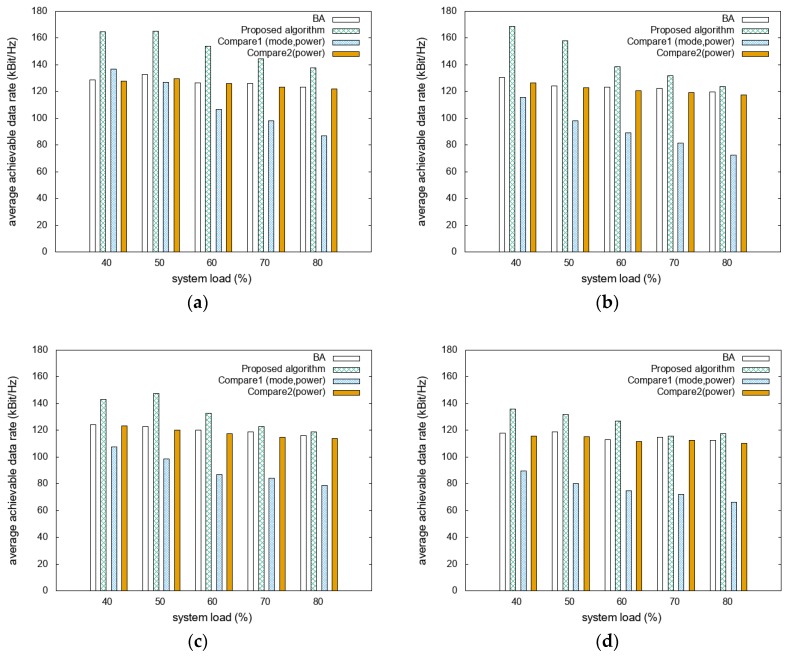
Comparison of average achievable data rate with various D2D distances and system loads: (**a**) 250 m with light traffic; (**b**) 350 m with light traffic; (**c**) 250 m with heavy traffic; and (**d**) 350 m with heavy traffic.

The average achievable data rates for the different D2D distance scenarios are compared as functions of system loads with different mobility scenarios, as shown in Figure 4. Figure 4a shows that the proposed algorithm has achievable data rates that are higher than those of BA, Compare1, and Compare2 by approximately 20%, 40%, and 22%, respectively. Figure 4b shows that the achievable data rate of the proposed algorithm is 60% higher than that of Compare1. Compared to BA and Compare2, it presents higher energy efficiency, with differences of approximately 16% and 19%, respectively. In Figure 4c, the proposed algorithm presents approximately 10%, 47%, and 13% higher achievable data rates compared to those of BA, Compare1, and Compare2, respectively. Figure 4d shows that the proposed algorithm outperforms BA, Compare1, and Compare2 by approximately 9%, 65%, and 11%, respectively. As the D2D distance increases, increasing the D2D communication ratio results in reduced energy consumption. However, increasing the D2D distance decreases the achievable data rates because the D2D mode communicates with the same transmission power even in degraded interference situations. In addition, communication without mode-selection algorithms, such as BA and Compare2, can select the transmission mode by simply considering the expected SINR, so it can perform better in terms of achievable data rate than algorithms with mode selection. The proposed algorithm achieved a higher achievable data rate even in such scenarios by adjusting the target SINR.

**Figure 5:**
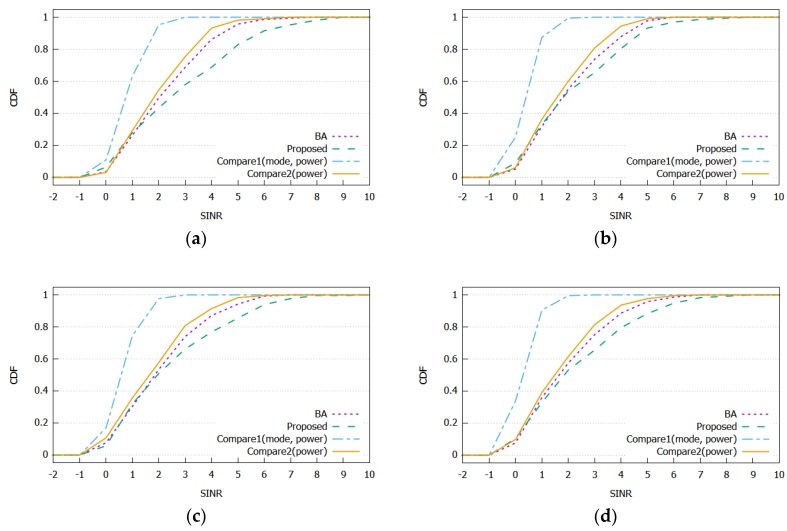
Comparison of signal-to-interference noise ratio (SINR) with various D2D distances and system loads: (**a**) 250 m with light traffic; (**b**) 350 m with light traffic; (**c**) 250 m with heavy traffic; and (**d**) 350 m with heavy traffic.

The cumulative distribution functions (CDFs) of SINR received by vehicles for different D2D distance thresholds are compared in Figure 5. Figure 5a–d shows the distribution of the received SINR in each algorithm. In all panels, the proposed algorithm has more vehicles that receive higher SINR compared to BA, Compare1, and Compare2. The proposed algorithm and Compare1 can select the communication mode and transmission power to maximize the system energy efficiency, allowing vehicles to select more D2D modes. It can increase spectral efficiency and reduce system energy consumption, but it can also decrease the received SINR. However, the proposed algorithm is designed to select the mode and transmission power to ensure the received SINR. Therefore, the proposed algorithm has more vehicles, receives higher SINR, and performs better with an achievable data rate compared to Compare1. The BA and Compare2 decide the communication mode based only on the expected SINR of the vehicle, so they can achieve higher performance in a longer D2D distance threshold.

**Figure 6:**
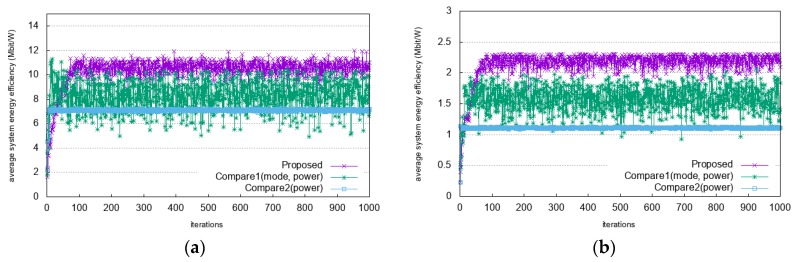
Convergence of system energy efficiency as a reward function with D2D distance = 350 m: (**a**) light traffic scenario; (**b**) heavy traffic scenario.

The comparisons of system energy efficiency as a reward function with an increasing number of learning iterations are shown in Figure 6. In Figure 6a it can be seen that as the number of learning iterations increases, the value of system energy efficiency stabilizes after 100 iterations. In heavy traffic scenario, as shown in Figure 6b, the value of the system energy efficiency stabilizes after 100 iterations. The system energy efficiency is overserved with a low value at the beginning of the learning iterations and increases until it reaches a stable point. This means that the proposed algorithm updates its policy optimally and converges faster and in a more stable manner than the other algorithms. The convergence time of the proposed algorithm is longer than those of Compare1 and Compare2. This is because the proposed algorithm has longer decision process and considers more states in decision process. It finally decides an action by comparing two recommended actions. In addition, it considers available resources and interference level as state to make a decision. However, Compare1 and Compare2 consider only interference level as state. Although the convergence time of the proposed algorithm becomes a little bit longer, the proposed algorithm shows better performance in terms of system energy efficiency.

To show the comparison with the optimal solution, the performance values are shown in Table 2 and Table 3. In Table 2, the average system energy efficiencies with system load variance are described. Based on the optimal solution, the proposed algorithm, BA, Compare1, and Compare2 obtain 44%, 20%, 35%, and 20% of the system energy efficiency, respectively. In Table 3, the average achievable data rate with system load variance is described. Based on the optimal solution, the proposed algorithm, BA, Compare1, and Compare2 obtain 79%, 68%, 50%, and 66% of the achievable data rate, respectively. This shows that the proposed algorithm performs closest to the optimal solution among other compared algorithms because it adopts the system energy efficiency and received SINR as rewards. However, as the system load increases, the differences in performance increase. This is because the interference increases according to increasing system load environment. The proposed algorithm simply models received SINR as whether the average SINR is greater than the target SINR to take account of the interference.

To evaluate and discuss the performance of the proposed algorithm, we compared it with the BA and the two compared algorithms. The proposed algorithm exhibited the best performance in terms of system energy efficiency, achievable data rate, and SINR in cases of increasing D2D distance thresholds. The proposed algorithm learned to maximize the system energy efficiency while ensuring achievable data rates. The system energy efficiency and achievable data rate have a trade-off relationship, so the proposed algorithm used Q-learning in two ways. To maximize the system energy efficiency, the proposed algorithm sets the system energy efficiency as a reward function of centralized Q-learning. In addition, to ensure an achievable data rate, the proposed algorithm sets the received SINR as a reward function of distributed Q-learning. This implies that the proposed algorithm can achieve the highest energy efficiency when compared to other algorithms. It can also guarantee QoS while increasing the efficiency of the resource and system energy.

## 6. Conclusions

We proposed a joint mode-selection and power-control algorithm with reinforcement learning to achieve energy optimization in vehicle networks. We defined and formulated the maximization problem for system energy efficiency, subject to SINR and outage probability constraints. We considered cellular and D2D communications coexisting in a single-cell environment and designed a Q-learning algorithm that made optimal transmission-mode-selection and power-control decisions by adjusting the target SINR. The Q-learning algorithm used centralized Q-learning in the BBU pool and decentralized Q-learning in the devices. To show that the proposed algorithm outperformed the other algorithms, we used a SUMO simulator to run various scenarios and compared system energy efficiencies, achievable data rates, and SINRs.

In real network environments, the system may not be fully aware of the channel state information. To solve this problem, clustering and sharing information among neighboring cells is necessary. In future work, we will consider a cell clustering and sharing method that provides optimal decisions to devices that handover nearby cells in environments with uncertain channel state information.

## Figures and Tables

**Table 1:** Parameters used in the simulation.

Parameter	Notation	Value
Noise power spectral density	N	−174 dBm/Hz
Total bandwidth	$W^{total}$	100 MHz
SINR constraint	$\gamma_{0}$	0.5 dBm
Maximum outage probability constraint	$\tau_{max}$	0.05
Minimum outage probability constraint	$\tau_{min}$	0.01
Circuit power of RRH	$\phi_{rrh}$	4.3 W
Slope of RRH	Δslope	4.0
Circuit power of fronthaul transceiver and switch	$\phi_{fronthaul}$	13 W
Power consumption per bit/s	ℓ	0.83 W
Transmission power of cellular device	$p_{k}^{c}$	23 dBm
Transmission power of RRH	$p_{tr}^{0}$	24 dBm

**Table 2:** Average system energy efficiencies with system load variance, D2D distance=350 m.

System Load (%)	40	50	60	70	80
Optimal	4.8414	5.2831	4.9594	5.4548	5.5733
BA	1.9857	1.0919	0.8479	0.7086	0.6553
Proposed	3.8026	2.5863	1.9223	1.5863	1.4909
Compare1	3.2371	1.9767	1.5212	1.2576	1.1491
Compare2	1.9429	1.0723	0.8184	0.6992	0.6376

**Table 3:** Average achievable data rate with system load variance, D2D distance=350 m.

System Load (%)	40	50	60	70	80
Optimal	183.1308	184.869	181.0795	180.4453	179.5396
BA	130.6982	124.3168	123.1323	122.5992	119.5717
Proposed	168.763	158.1163	138.6988	131.8081	123.8312
Compare1	115.4847	98.18192	88.934	81.2835	72.47508
Compare2	126.4065	122.9883	120.3971	119.4175	117.3408
